# Supplementary material for: Machine learning prediction of connectivity, biodiversity and resilience in the Coral Triangle
Source: Commun Biol. 2022 Dec 10;5:1359. doi: 10.1038/s42003-022-04330-8 (PMC9741626; doi:10.1038/s42003-022-04330-8)
Supplement: Supplementary file 2 — Supplemental Material [file 42003_2022_4330_MOESM2_ESM.pdf]

Supplementary Materials for

Machine Learning prediction of connectivity, biodiversity and resilience in the Coral Triangle

Lyuba Novi, Annalisa Bracco*

*Corresponding author. Email: abracco@gatech.edu

This PDF file includes:

Supplementary Figs. 1 to 15
Supplementary Table 1

The MATLAB codes to recreate the Supplementary Figs. 1 to 15 are publicly available through figshare (https://doi.org/10.6084/m9.figshare.21587199.v1).

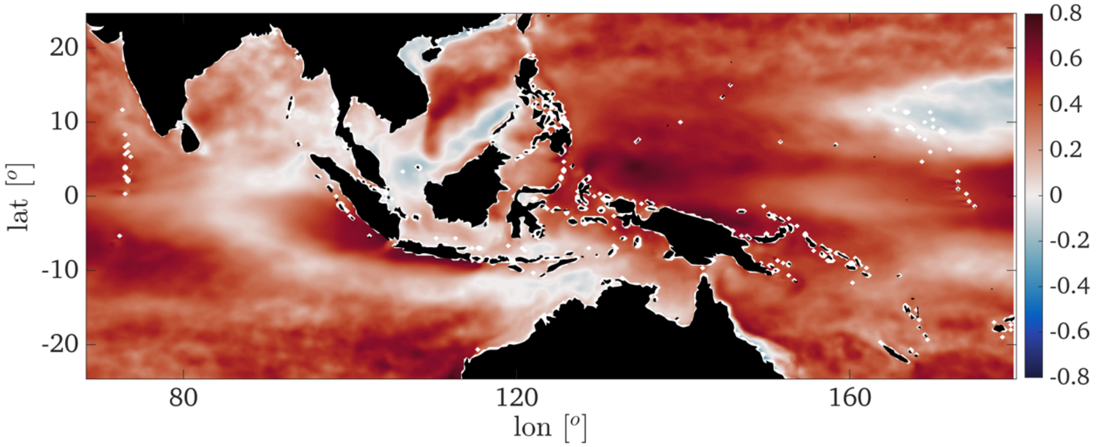

Supplementary Fig. 1.

SSH anomalies and SST anomalies correlation. Time correlation between SSH anomalies and SST anomalies over 1993-2017. SSH and SST data are from the GLORYS12V1 reanalysis product and are remapped at 1/3°resolution.

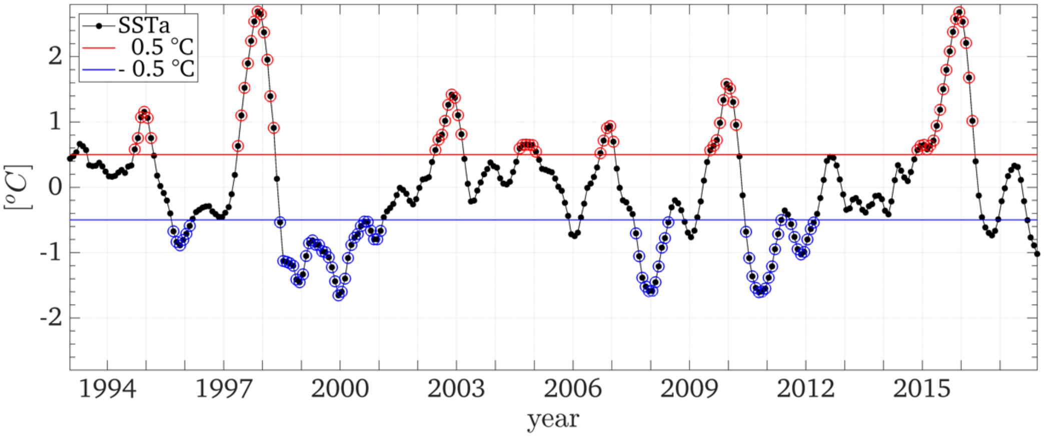

Supplementary Fig. 2.

SST anomalies 3-month moving mean over ENSO 3.4 region (ONI index). Red (blue) circles indicate 5 or more consecutive months with SST anomalies above (below) 0.5°C and identify El Niño (La Niña) years over January 1993 - December 2017.

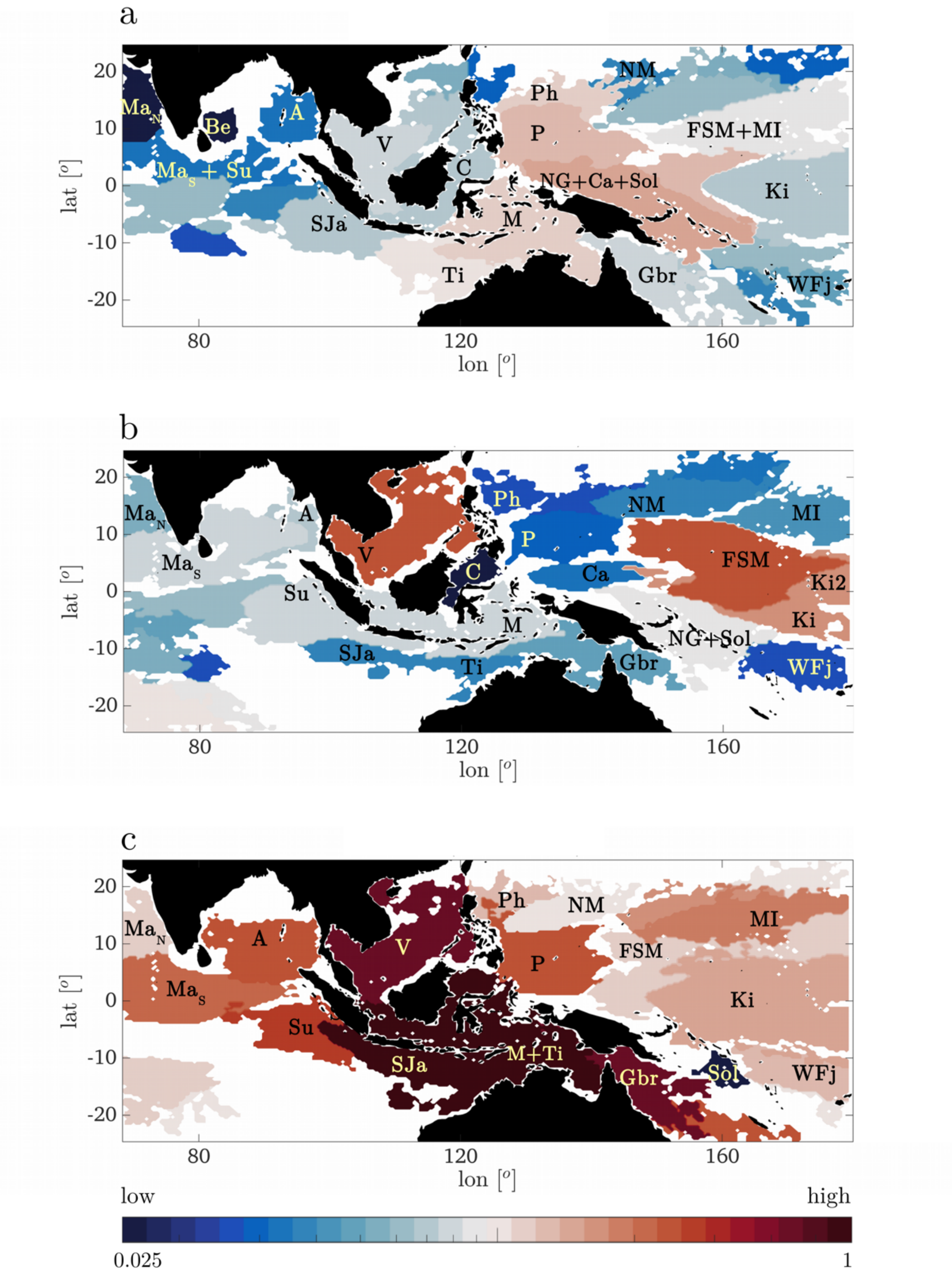

Supplementary Fig. 3.

Domain strengths for $\tau_{max} = 1$: Domains over the period 1993-2017 in the CT and Indian Ocean for $\tau_{max} = 1$ months for aggregated (a) neutral, (b) El Niño, and (c) La Niña years, colored according to their strength value. In each period, domains with strength below the 20th percentile (computed for that period) are not shown. Strengths are normalized by 6×10^6 and the color scale is logarithmic.

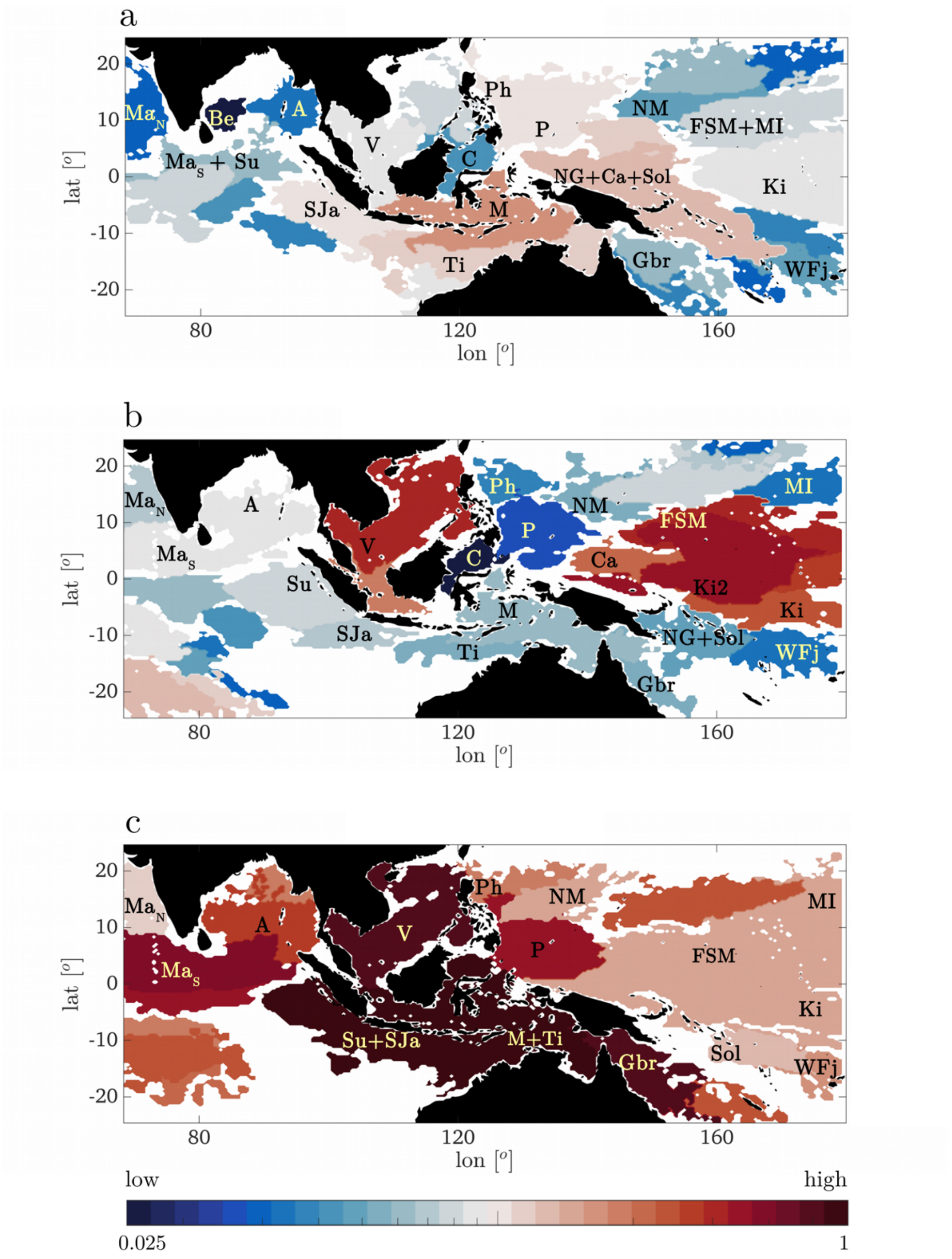

Supplementary Fig. 4.
Domain strengths for $\tau_{max} = 3$. Domains over the period 1993-2017 in the CT and Indian Ocean for $\tau_{max} = 3$ months for aggregated (a) neutral, (b) El Niño, and (c) La Niña years, colored according to their strength value. In each period, domains with strength below the 20th percentile (computed for that period) are not shown. Strengths are normalized by 6×10^6, and the color scale is logarithmic.

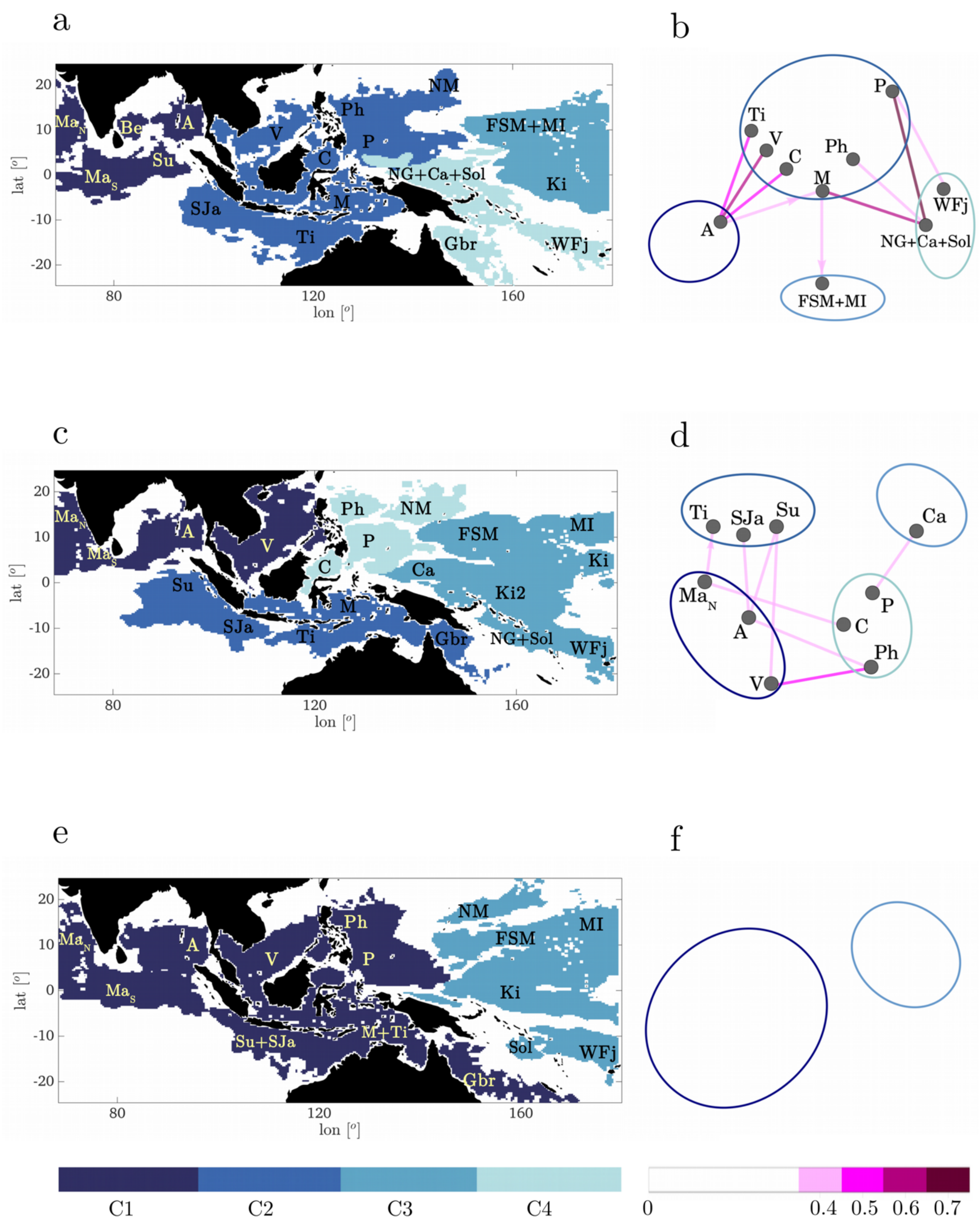

a
b
c
d
e
f
lat [o]
lon [o]
80
120
160
20
10
0
-10
-20
Ma_N
Ma_S
Be
A
Su
V
Ph
P
C
M
NM
SJa
Ti
FSM+MI
NG+Ca+Sol
Ki
Gbr
WFj
FSM
MI
Ca
Ki2
NG+Sol
Sol
Su+SJa
M+Ti
C1
C2
C3
C4
0
0.4
0.5
0.6
0.7

Supplementary Fig. 5

Community structure. Supercommunities reported with different colors (a, c, e), with connections between them colored according to their strength (correlation coefficient) (b, d, f). Only nodes and links connecting different communities are shown. (a, b) Neutral, (c, d) El Niño, (e, f) La Niña years.

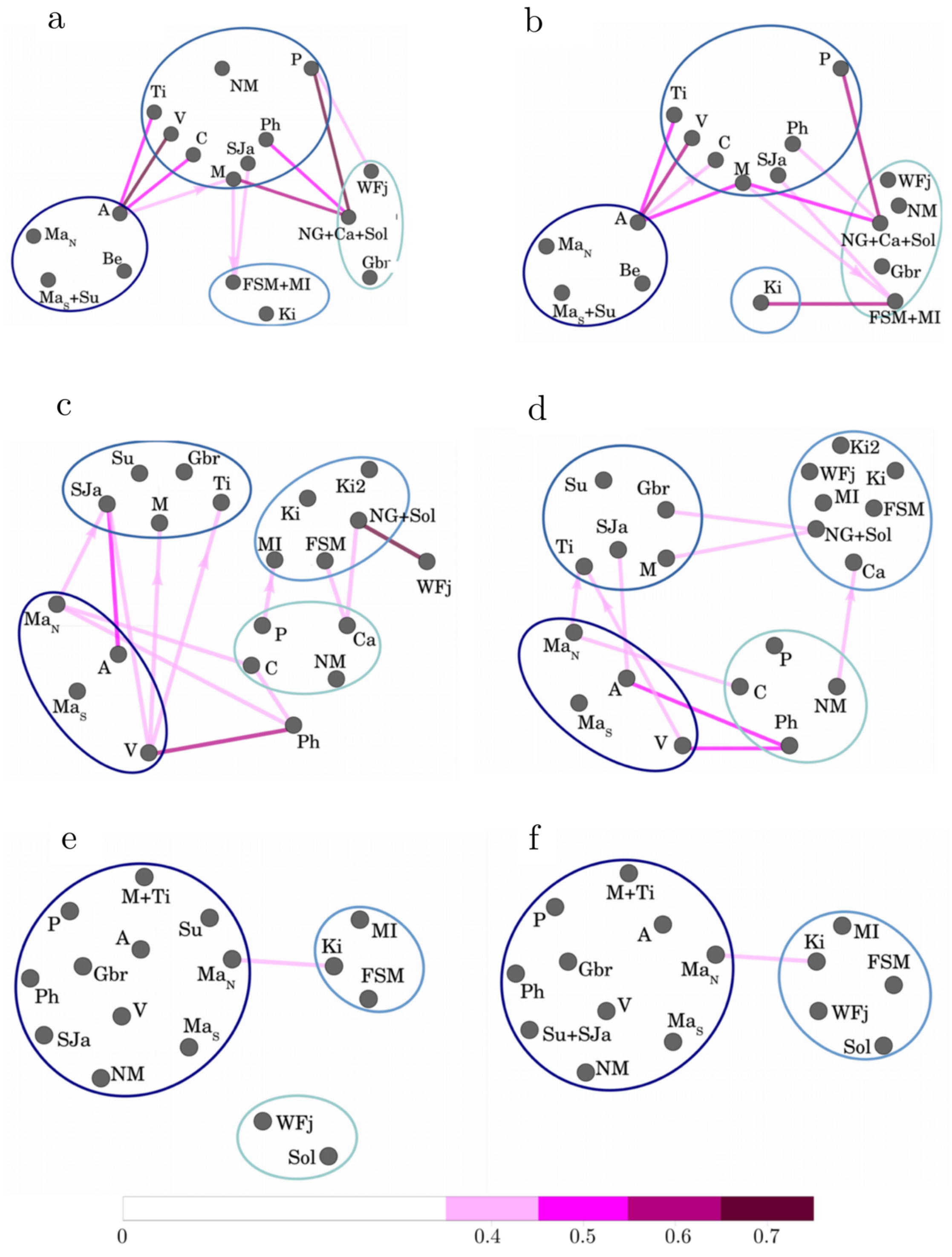

Supplementary Fig. 6.

Supercommunities and their connections for $\tau_{max} = 1$(a, c, e) and $\tau_{max} = 3$ (b, d, f). Links are colored according to their correlation coefficient. Only links connecting different communities are shown. (a, b) Neutral, (c, d) El Niño, (e, f) La Niña years. At $\tau_{max} = 1$, for El Niño conditions, ecoregions Ph and WFj are excluded from the communities because of the shorter PLD, while WFj and Sol are isolated into a small, separated community in La Niña years.

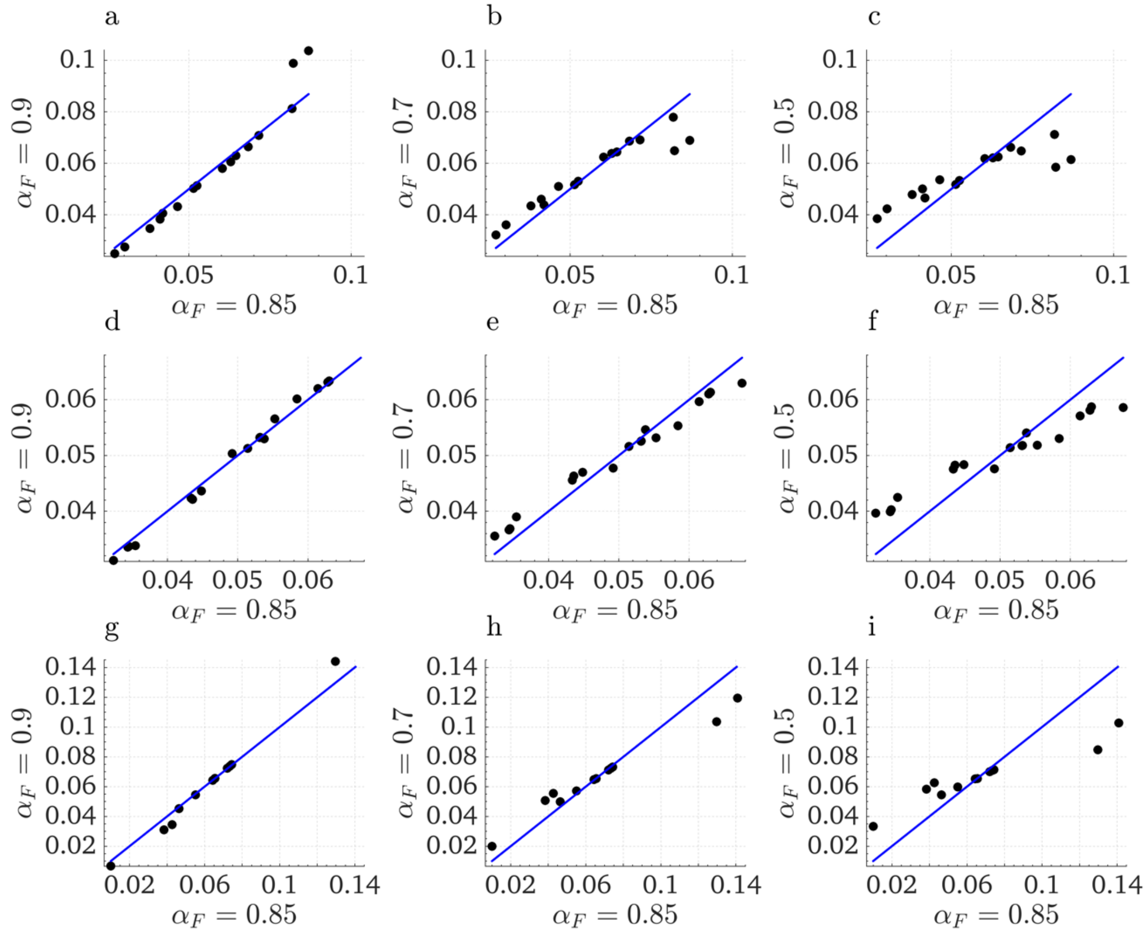

Supplementary Fig. 7.

Robustness of the Page Rank Centrality to the choice of the damping factor for $\tau_{max} = 2$. (a), (b), (c) Neutral years; (d), (e), (f) El Niño years; (g), (h), (i) La Niña years. The blue line in each panel is a bisector. The robustness has been similarly verified for $\tau_{max} = 1$ and $\tau_{max} = 3$.

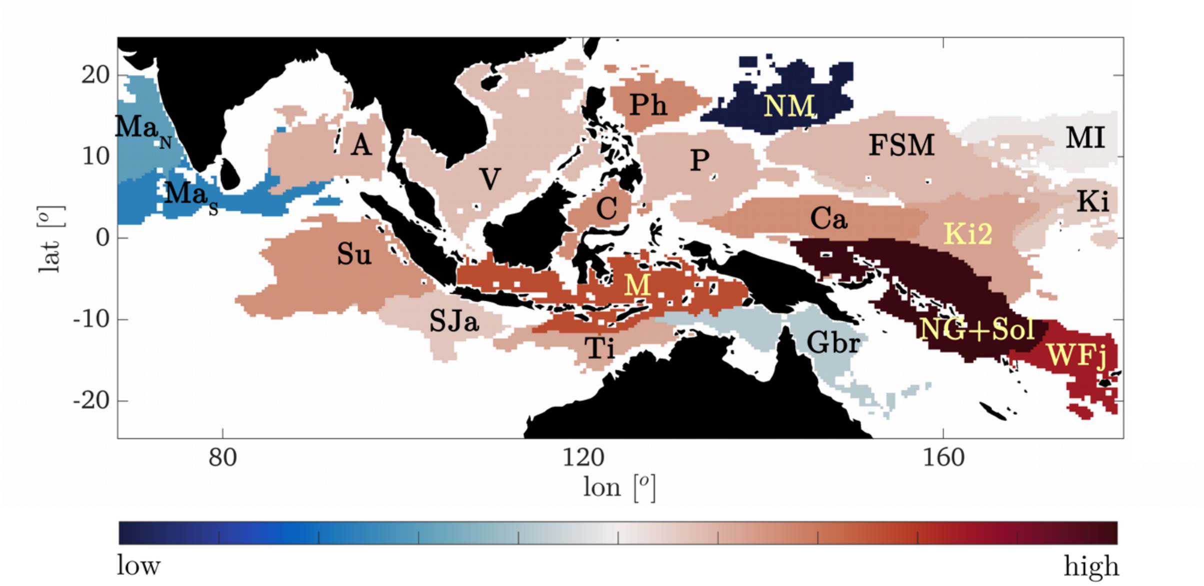

Supplementary Fig. 8. Estimated Biodiversity Score over 1993-2017 averaged over ENSO phases. Domains found during El Nino years, colored according to their Average Biodiversity Score. The color scale is linear (low = 0.03, high = 0.08), and the Biodiversity Score is averaged among the values obtained for El Nino, La Nina and Neutral-years cases.

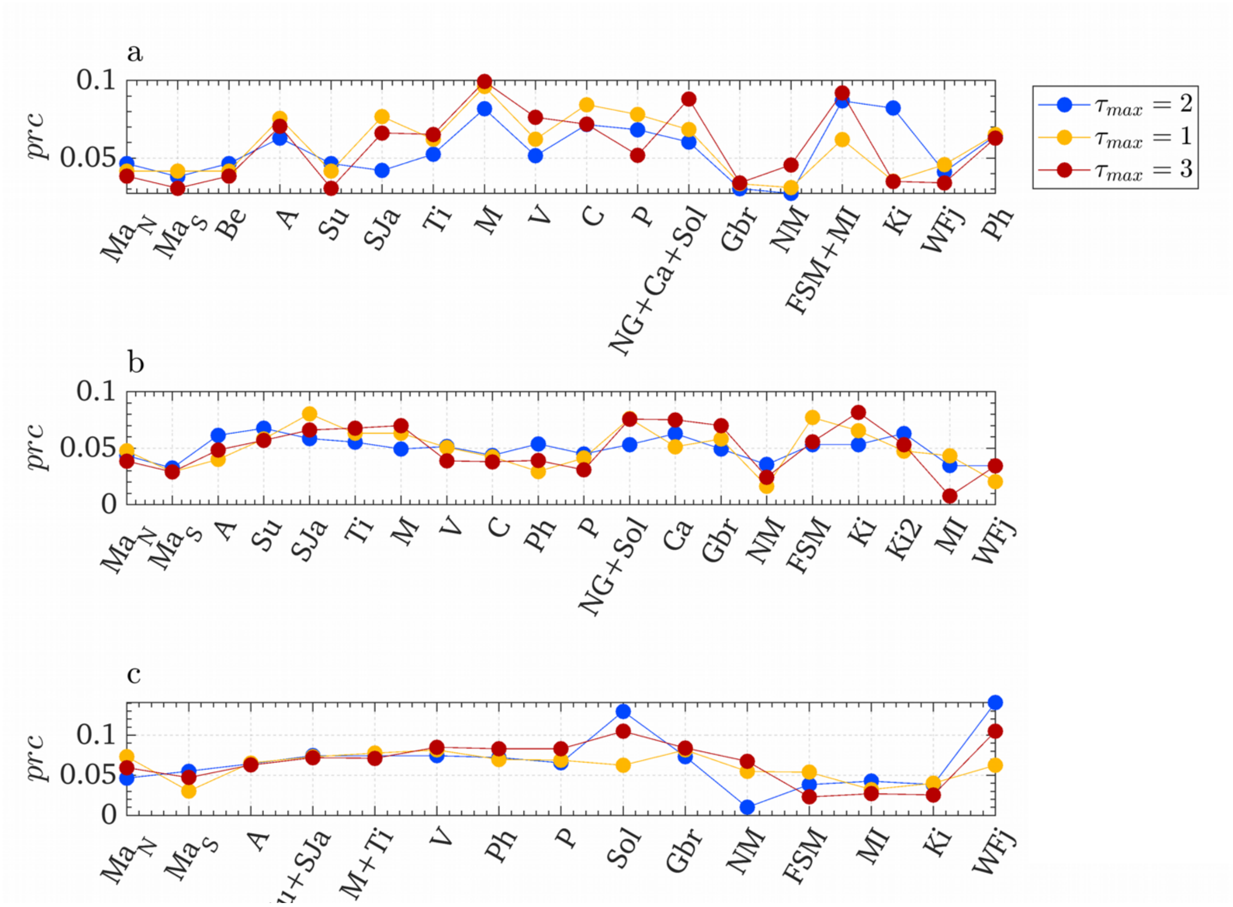

Supplementary Fig. 9.

Robustness of the Page Rank Centrality with τ_{max} changes. (a) Neutral years, (b) El Niño years, (c) La Niña years for damping factor 0.85. A similar agreement between τ_{max} = 1, 2 and 3 was found also for damping factors 0.5, 0.7 and 0.9 (not shown).

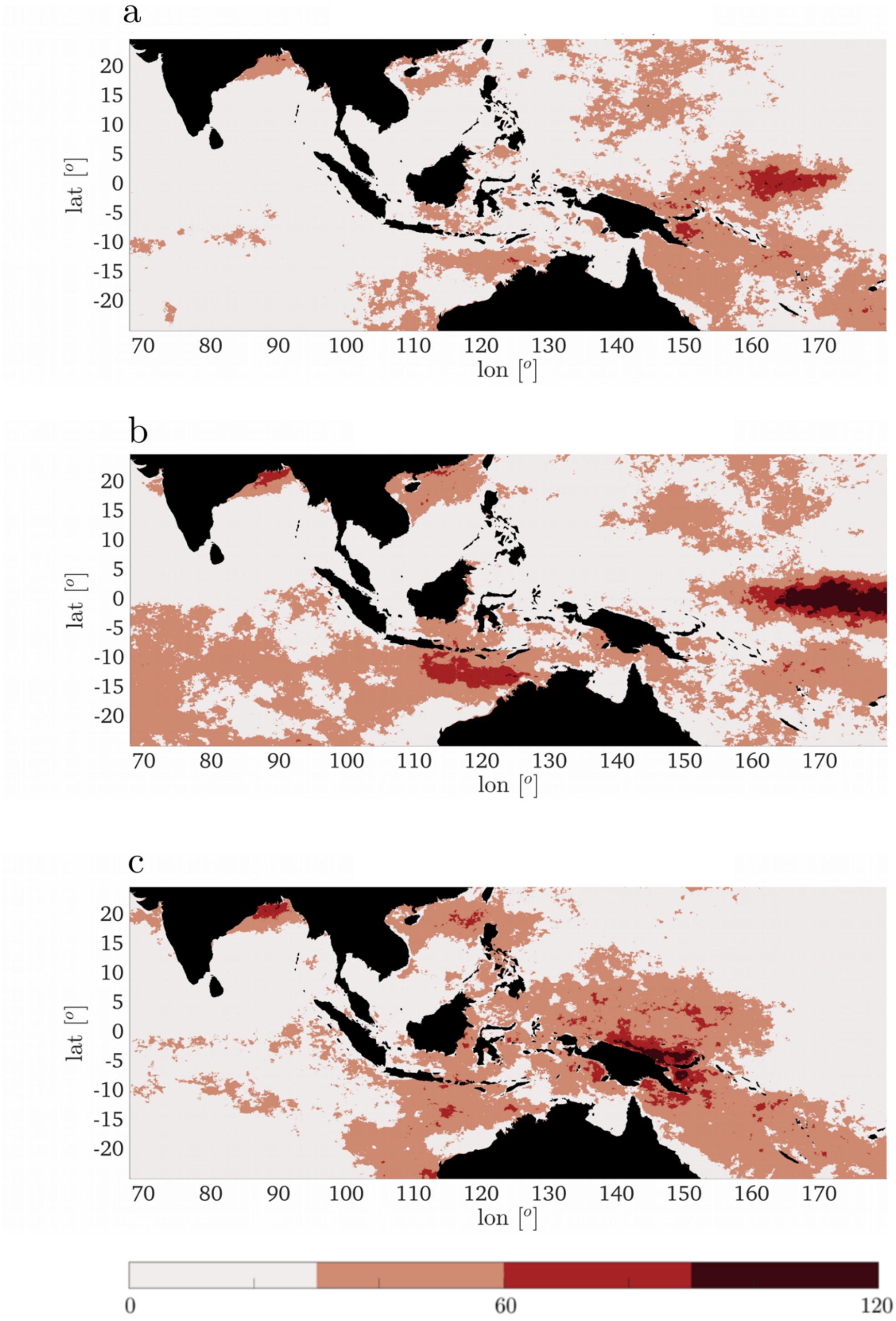

Supplementary Fig. 10.
Cumulative bleaching area alert during different ENSO phases. Time cumulative values of *baa* “Watch”, “Alert Level 1” and “Alert level 2” for (a) Neutral years, (b) El Niño years and (c) La Niña years. Composites of bleaching alert area monthly maximum (*baa*) are from the NOAA Coral Reef Watch's (CRW) Version 3.1 daily 5km (0.05 degree) satellite coral bleaching heat

stress monitoring products. El Niño conditions lead to higher *t.s.baa* values especially over the equatorial west Pacific islands, the southern CT, and the southern Indian Ocean, while some other areas result less stressed compared to neutral years, such as Papua New Guinea and the Great Barrier Reef. In La Niña years, *t.s.baa* values are higher than those in neutral years over much of the CT, Papua New Guinea, Philippines, Caroline Islands and Palau. Bleaching stress in the southern Indian Ocean during La Niña years is limited to the eastern part of the basin. Despite several areas displaying strong *t.s.baa* differences depending on the ENSO phase, the South China Sea or the coasts of Sri Lanka are mostly insensitive to ENSO variability.

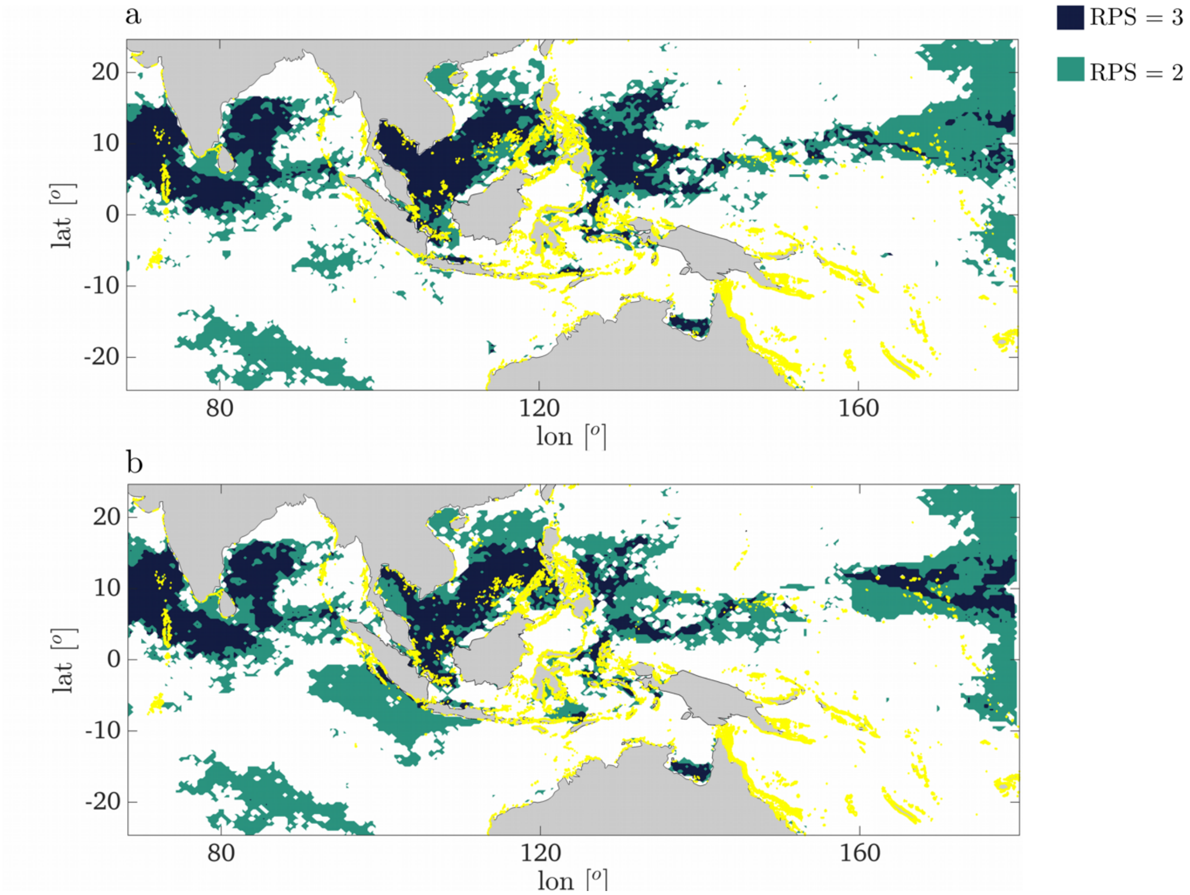

Supplementary Fig. 11.
RPS robustness with τ_{max}. (a) $\tau_{max} = 1$ and (b) $\tau_{max} = 3$.

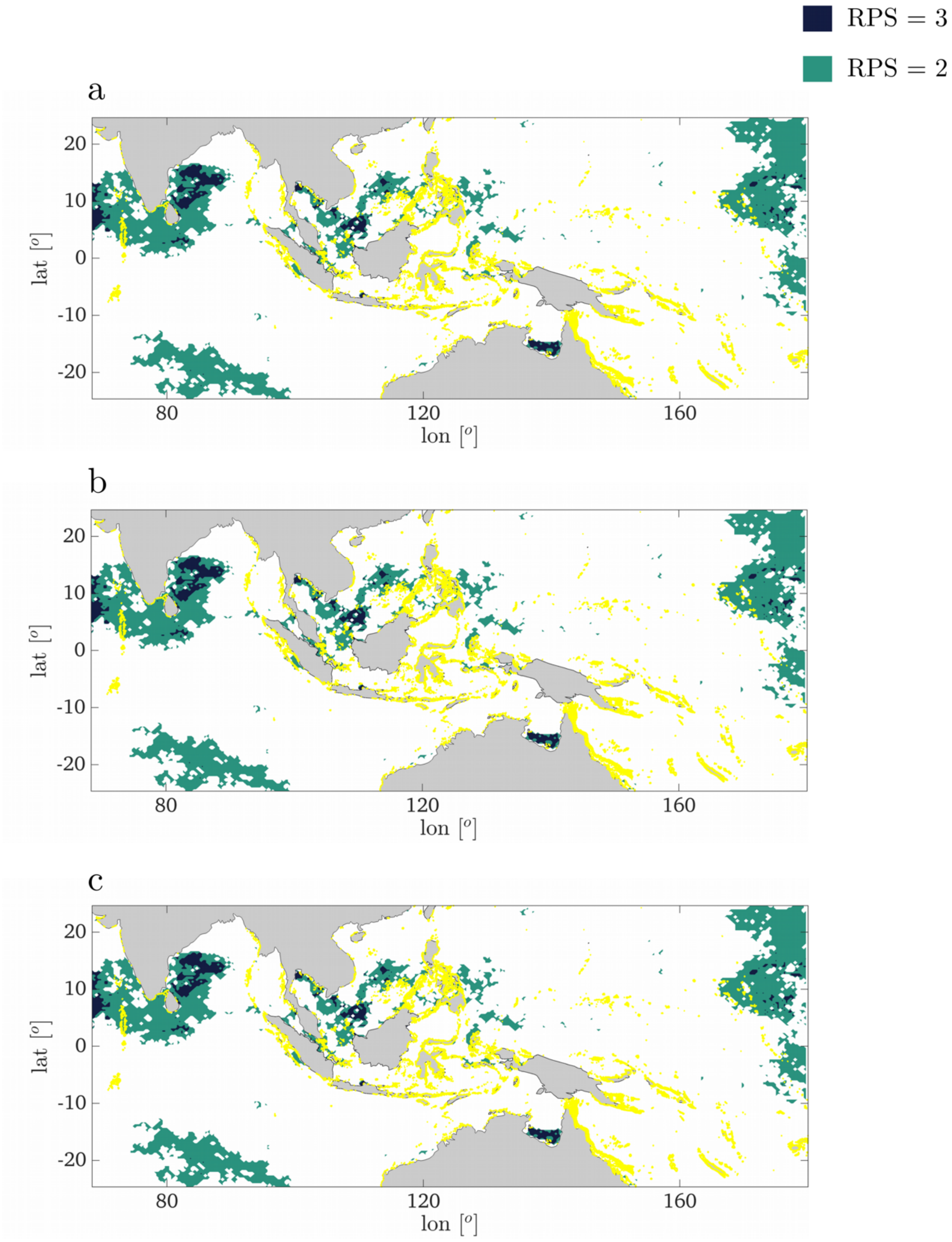

Supplementary Fig. 12.
RPS calculated considering only t.s.baa. (a) $\tau_{max} = 2$, (b) $\tau_{max} = 1$, (c) $\tau_{max} = 3$.

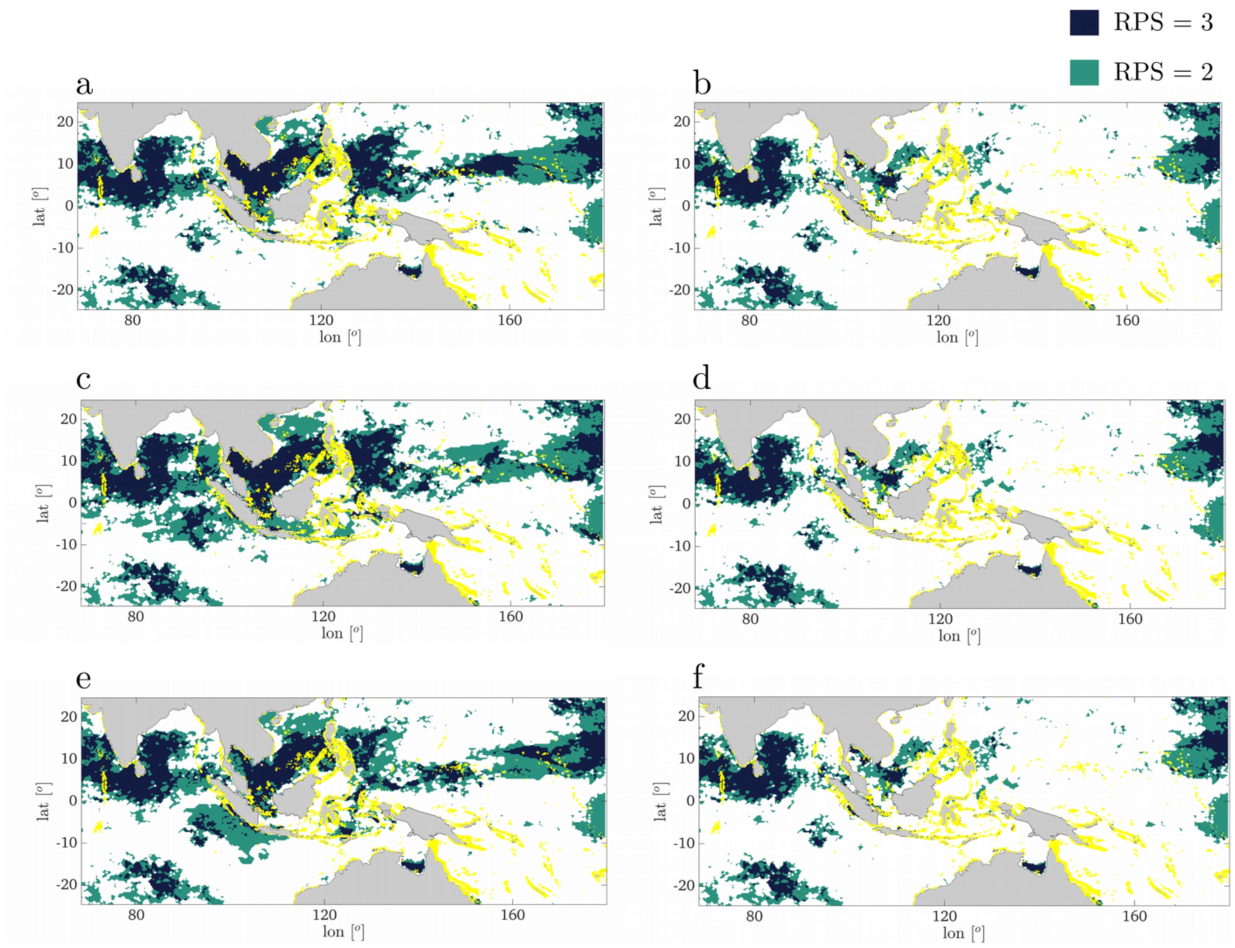

Supplementary Fig. 13.

RPS computed using Degree Heating Weak instead of Bleaching Alert Area. (a) $\tau_{max} = 1$, (c) $\tau_{max} = 2$ and (e) $\tau_{max} = 3$. Right column: RPS calculated using only t.s. DHW. (b) $\tau_{max} = 1$, (d) $\tau_{max} = 2$ and (f) $\tau_{max} = 3$.

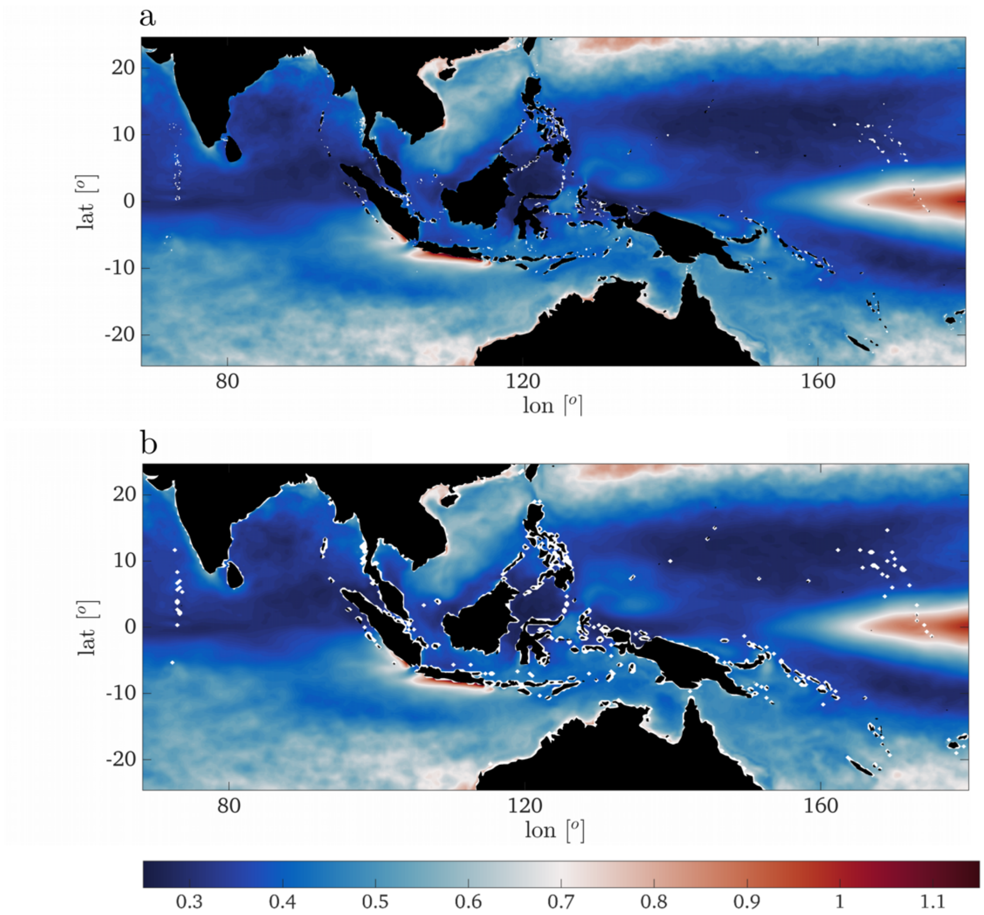

Supplementary Fig. 14.
Robustness to SST anomalies resolution upscaling. The time standard deviation (°C) of SST anomalies from the GLORYS12V1 reanalysis product is computed with data at (a) 1/12° resolution and (b) 1/3°resolution. This comparison shows that the lower resolution captures the same spatial and temporal variability for the region under consideration, being more than sufficient to resolve the Rossby deformation radius at these low latitudes.

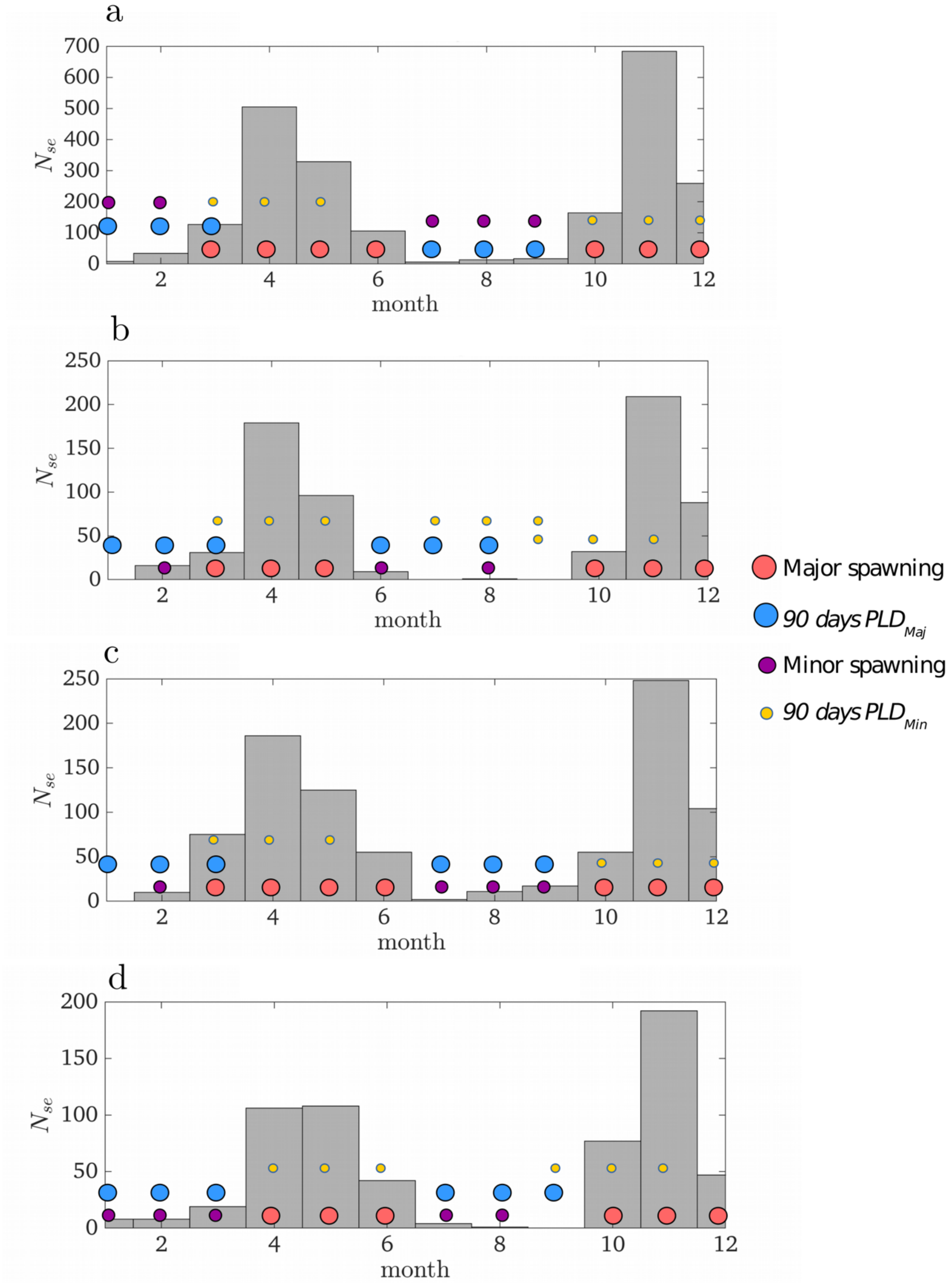

Supplementary Fig. 15.
Monthly distribution of coral spawning events. Monthly distribution of the number of spawning events (N_{se}) in our study area, as obtained by the CSD collection over (a) all years from 1993 to 2017, (b) aggregated neutral years, (c) aggregated El Niño years, and (d) aggregated La Niña years. Red circles indicate major spawning months, purple circles indicate

minor spawning months; months with potential larval availability for a 60-day PLD after a last minor (major) spawning months are indicated by yellow (turquoise) circles.

Supplementary Table 1.

El Niño years	La Niña years	Neutral years
April 1994 – March 1995<br>April 1997 – March 1998<br>April 2002 – March 2003<br>April 2004 – March 2005<br>April 2006 – March 2007<br>April 2009 – March 2010<br>April 2014 – March 2015<br>April 2015 – March 2016	April 1995 – March 1996<br>April 1998 – March 1999<br>April 1999 – March 2000<br>April 2000 – March 2001<br>April 2007 – March 2008<br>April 2010 – March 2011<br>April 2011 – March 2012<br>April 2016 – March 2017	April 1993 – March 1994<br>April 1996 – March 1997<br>April 2001 – March 2002<br>April 2003 – March 2004<br>April 2005 – March 2006<br>April 2008 – March 2009<br>April 2012 – March 2013<br>April 2013 – March 2014

Selected years in each ENSO phase.
