# Supplementary material for: Machine learning prediction of connectivity, biodiversity and resilience in the Coral Triangle
Source: Commun Biol. 2022 Dec 10;5:1359. doi: 10.1038/s42003-022-04330-8 (PMC9741626; doi:10.1038/s42003-022-04330-8)
Supplement: Supplementary file 3 — Reporting Summary [file 42003_2022_4330_MOESM3_ESM.pdf]

nature portfolio

Corresponding author(s): Annalisa Bracco

Last updated by author(s): 11/17/2022

Reporting Summary

Nature Portfolio wishes to improve the reproducibility of the work that we publish. This form provides structure for consistency and transparency in reporting. For further information on Nature Portfolio policies, see our Editorial Policies and the Editorial Policy Checklist.

Statistics

For all statistical analyses, confirm that the following items are present in the figure legend, table legend, main text, or Methods section.

n/a	Confirmed	
☒	☐	The exact sample size (*n*) for each experimental group/condition, given as a discrete number and unit of measurement
☒	☐	A statement on whether measurements were taken from distinct samples or whether the same sample was measured repeatedly
☒	☐	The statistical test(s) used AND whether they are one- or two-sided<br>*Only common tests should be described solely by name; describe more complex techniques in the Methods section.*
☒	☐	A description of all covariates tested
☒	☐	A description of any assumptions or corrections, such as tests of normality and adjustment for multiple comparisons
☐	☒	A full description of the statistical parameters including central tendency (e.g. means) or other basic estimates (e.g. regression coefficient) AND variation (e.g. standard deviation) or associated estimates of uncertainty (e.g. confidence intervals)
☒	☐	For null hypothesis testing, the test statistic (e.g. *F*, *t*, *r*) with confidence intervals, effect sizes, degrees of freedom and *P* value noted<br>*Give P values as exact values whenever suitable.*
☒	☐	For Bayesian analysis, information on the choice of priors and Markov chain Monte Carlo settings
☐	☒	For hierarchical and complex designs, identification of the appropriate level for tests and full reporting of outcomes
☐	☒	Estimates of effect sizes (e.g. Cohen's *d*, Pearson's *r*), indicating how they were calculated

Our web collection on statistics for biologists contains articles on many of the points above.

Software and code

Policy information about availability of computer code

Data collection	N/A
Data analysis	δ-MAPS software (java version) can be found at https://zenodo.org/record/7320416#.Y3LesoLMLdo, https://doi.org/10.5281/zenodo.7320416

For manuscripts utilizing custom algorithms or software that are central to the research but not yet described in published literature, software must be made available to editors and reviewers. We strongly encourage code deposition in a community repository (e.g. GitHub). See the Nature Portfolio guidelines for submitting code & software for further information.

Data

Policy information about availability of data

All manuscripts must include a data availability statement. This statement should provide the following information, where applicable:

- Accession codes, unique identifiers, or web links for publicly available datasets
- A description of any restrictions on data availability
- For clinical datasets or third party data, please ensure that the statement adheres to our policy

The analyzed data (SST and SSH) are obtained from the Global Ocean Physics Reanalysis GLORYS12V1 1/12° product35 MERCATOR GLORYS12V1 (global-reanalysis-001-030-monthly), available with free access through the E.U. Copernicus Marine Service Information (CMEMS) portal (https://doi.org/10.48670/moi-00021). The coral spawning data are from the "Coral Spawning Database" available at https://doi.org/10.25405/data.ncl.13082333.v1 . Composites of monthly

maximum bleaching alert area are from the NOAA Coral Reef Watch's (CRW) Version 3.1 heat stress monitoring products at daily frequency and 0.05o degree resolution, available at https://coralreefwatch.noaa.gov/product/5km/.

Human research participants

Policy information about studies involving human research participants and Sex and Gender in Research.

Reporting on sex and gender	N/A
Population characteristics	N/A
Recruitment	N/A
Ethics oversight	N/A

Note that full information on the approval of the study protocol must also be provided in the manuscript.

Field-specific reporting

Please select the one below that is the best fit for your research. If you are not sure, read the appropriate sections before making your selection.

☐ Life sciences ☐ Behavioural & social sciences ☒ Ecological, evolutionary & environmental sciences

For a reference copy of the document with all sections, see nature.com/documents/nr-reporting-summary-flat.pdf

Ecological, evolutionary & environmental sciences study design

All studies must disclose on these points even when the disclosure is negative.

Study description	This is an application of Machine Learning tools to explore how sea surface temperature data can be used to explore connectivity at tropical, equatorial and extra-tropical latitudes. All data used are publicly available and the software is available through GiTHUB
Research sample	N/A
Sampling strategy	N/A
Data collection	N/A
Timing and spatial scale	1993-2017; 25N-25S; 65E-180E
Data exclusions	N/A
Reproducibility	We repeated each connectivity calculation considering 1, 2 or 3 months for average pelagic larval duration and verified that results are not only highly statistically significant, but also unaffected by the choice of slightly different parameters
Randomization	N/A
Blinding	N/A

Did the study involve field work? ☐ Yes ☒ No

Reporting for specific materials, systems and methods

We require information from authors about some types of materials, experimental systems and methods used in many studies. Here, indicate whether each material, system or method listed is relevant to your study. If you are not sure if a list item applies to your research, read the appropriate section before selecting a response.

Materials & experimental systems

n/a	Involved in the study	
☒	☐	Antibodies
☒	☐	Eukaryotic cell lines
☒	☐	Palaeontology and archaeology
☒	☐	Animals and other organisms
☒	☐	Clinical data
☒	☐	Dual use research of concern

Methods

n/a	Involved in the study	
☒	☐	ChIP-seq
☒	☐	Flow cytometry
☒	☐	MRI-based neuroimaging
